# Unicentric report on thoracoscopic surgery 7 cases for congenital pulmonary airway malformation combined with ipsilateral mediastinal bronchogenic cyst in children

**DOI:** 10.1186/s12893-026-03617-5

**Published:** 2026-03-05

**Authors:** Huashan Zhao, Shumin Zhao, Yunpeng Zhai, Rui Guo, Gang Shen, Hongxiu Xu, Sai Huang, Shisong Zhang

**Affiliations:** 1 Department of Thoracic and Oncological Surgery, Children’s Hospital Affiliated to Shandong University, Jinan, China; 2 Department of Thoracic and Oncological Surgery, Children’s Hospital Affiliated to Shandong University(Jinan Children’s Hospital), 23976 Jingshi Road, Huaiyin District, Jinan 250022 China; 3 Central Hospital Affiliated of Shandong First Medical University (Jinan Central Hospital), Jinan, China

**Keywords:** Thoracoscopic surgery, Children, Congenital pulmonary airway malformation (CPAM), Bronchogenic cyst, Ipsilateral mediastinum

## Abstract

**Objective:**

To evaluate the feasibility of thoracoscopic surgery for congenital pulmonary airway malformation (CPAM) with ipsilateral mediastinal bronchogenic cysts in children.

**Methods:**

From January 2019 to January 2025, our medical center performed a total of 462 surgeries on children with CPAM. Among them, 7 children who were diagnosed with CPAM with bronchogenic cysts underwent thoracoscopic surgery. A three-port approach via the lateral chest was adopted. Patients were placed in a lateral decubitus position (healthy side down). The observation hole is located at the junction of the scapular line and the 5th rib space, while the surgical hole is created using the 5-millimeter cannula through the endoscopic triangular method.CO₂ pneumothorax was maintained at 4 mmHg (flow rate: 2 L/min), adjusted intraoperatively as needed. Surgical incisions were primarily designed for pulmonary resection, prioritizing CPAM excision followed by bronchogenic cyst removal. Piecemeal resection was performed when necessary to avoid severe tracheal complications.

**Results:**

Among the 7 cases of CPAM with bronchogenic cysts, 5 were type II (bronchiolar) and 2 were type III (bronchiolar/alveolar). All procedures were successfully completed thoracoscopically without conversion to open surgery. Operative time ranged from 90 to 191 min (median: 110 min), with intraoperative blood loss of 3–15 mL (median: 10 mL). Chest tubes were placed in all cases for 3–5 days (median: 4 days). During follow-up (median: 29 months; range: 2–59 months), no disease recurrence was observed on chest CT.

**Conclusion:**

CPAM in children may coexist with bronchogenic cysts. Preoperative imaging should be meticulously correlated with intraoperative findings to avoid missed diagnoses. Thoracoscopic surgery is a method that can be attempted for treating CPAM in children with ipsilateral mediastinal bronchogenic cysts.

**Supplementary Information:**

The online version contains supplementary material available at 10.1186/s12893-026-03617-5.

Congenital Pulmonary Airway Malformation (CPAM) has a relatively low clinical prevalence, and its co-occurrence with other diseases is even rarer [1]. The combination of CPAM and bronchogenic cysts represents an exceedingly rare congenital pulmonary anomaly in pediatric thoracic surgery, with only sporadic case reports documented in the literature [2–4]. From January 2019 to January 2025, our medical center performed a total of 462 surgeries on children with CPAM(There were 503 cases of CPAM patients). Among them, 7༈1.39%,7/503) children who were diagnosed with CPAM with bronchogenic cysts underwent thoracoscopic surgery. The clinical details are reported as follows.

## Clinical data and methods

### General information

The cohort included 7 patients (5 males, 2 females), aged from 4 months and 3 days to 5 years and 7 months (median: 7 months and 13 days). Prenatal ultrasound identified congenital pulmonary cystic lesions in 6 cases, while 1 case was diagnosed postnatally by chest CT due to cough and pneumonia, revealing congenital pulmonary airway malformation (CPAM) combined with bronchogenic cyst. Preoperative CT confirmed pulmonary congenital anomalies with ipsilateral mediastinal cysts in all cases (left side: 1; right side: 6). Demographic and clinical characteristics are summarized in Table 1.

**Table 1:** Presents the demographic characteristics of the seven pediatric cases.

Case	Gender	Age (Months)	Lesion Location	Clinical Presentation	Preoperative CT Detection of Mediastinal Cyst	Size of Mediastinal Cyst on CT (cm)
1	Male	4	Right	Detected during prenatal ultrasound at 23 weeks of gestation	Yes	1.2 x 1.6 x 1.3
2	Male	6	Right	Detected during prenatal ultrasound at 28 weeks of gestation	Yes	1.8 x 1.3 x 1.0
3	Male	7	Right	Detected during prenatal ultrasound at 23 weeks of gestation	Yes	1.8 x 1.5 x 1.0
4	Female	67	Right	Detected on chest CT due to cough lasting 20 days	Yes	4.1 x 2.1 x 2.6
5	Female	7	Left	Detected during prenatal ultrasound at 22 weeks of gestation	Yes	1.1 x 1.1 x 0.6
6	Male	5	Right	Detected during prenatal ultrasound at 24 weeks of gestation	Yes	1.2 x 1.2 x 1.4
7	Male	20	Right	Detected during prenatal ultrasound at 28 weeks of gestation	Yes	1.1 x 1.1 x 0.6

Inclusion criteria:1.Preoperative CT-confirmed pulmonary congenital anomaly with mediastinal cyst.2.Absence of preoperative respiratory infection.3.No surgical contraindications.4.Planned thoracoscopic surgery.

Our medical center treated a total of 462 children who underwent CPAM surgery without infection from January 2019 to January 2025. According to the inclusion criteria, we screened out 7 cases from the 462 cases. We analyzed and summarized the clinical treatment of these 7 children.

### Surgical technique

All procedures were performed under general anesthesia with one-lung ventilation. A three-port fully thoracoscopic approach was adopted via the lateral chest. Patients were placed in a lateral decubitus position (healthy side down). The observation port was positioned at the intersection of the scapular line and the 5th intercostal space, while operative ports were established based on surgical needs following the triangulation (all ports used 5-mm trocars). CO₂ pneumothorax was maintained at 4 mmHg pressure and 2 L/min flow, adjusted intraoperatively as needed.

According to preoperative CT and intraoperative findings, either non-anatomical wedge resection or segmentectomy was performed. The lesion margins were demarcated with an electrocautery hook, followed by dissection of the arterial, bronchial, and venous structures using the hook and dissecting forceps.

When the congenital pulmonary airway malformation (CPAM) is confined to a specific pulmonary segment, segmentectomy is performed. The segmentectomy follows the standard sequence of artery (A) → vein (V) → bronchus (B). During the operation, the target bronchus is clamped to verify the resected pulmonary segment. The inter - segmental plane is identified, and the diseased pulmonary segment is resected according to the A→V→B sequence. After the operation, a pathological examination of the surgical wound is conducted to confirm the absence of residual lesions at the resection margin.

If the CPAM lesion is small and well - circumscribed, non - anatomical wedge resection of the lung is carried out. The lung surface is marked with an electric hook. Based on the CT scan, it is determined whether the intrapulmonary boundary of the CPAM lesion is defined by veins, arteries, or bronchi. Then, the lesion is completely resected by separating along the corresponding pulmonary boundary of the lesion. After the operation, a pathological examination of the surgical wound is performed to ensure the absence of residual lesions at the resection margin.

Since CPAM is a benign lesion, extended resection is generally not required. Completely enucleating the lesion along its margin may be the ideal outcome we pursue in surgery.

Lung parenchyma was sealed with Ligasure, and the resection surface was reinforced with 4 − 0 PDS sutures to minimize air leakage.

**Fig. 1:**
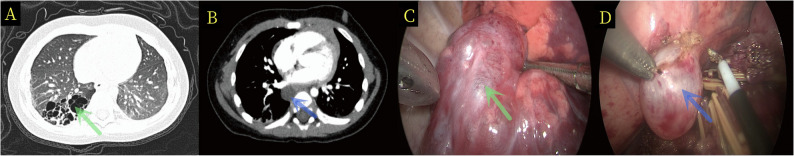
Displays the preoperative CT images and intraoperative thoracoscopic findings of Case 1. The congenital pulmonary airway malformation (CPAM) lesions are indicated by green arrows in Fig. 1**A** and **C**, while the bronchogenic cyst lesions are marked by blue arrows in Fig. 1**B** and **D**

**Fig. 2:**
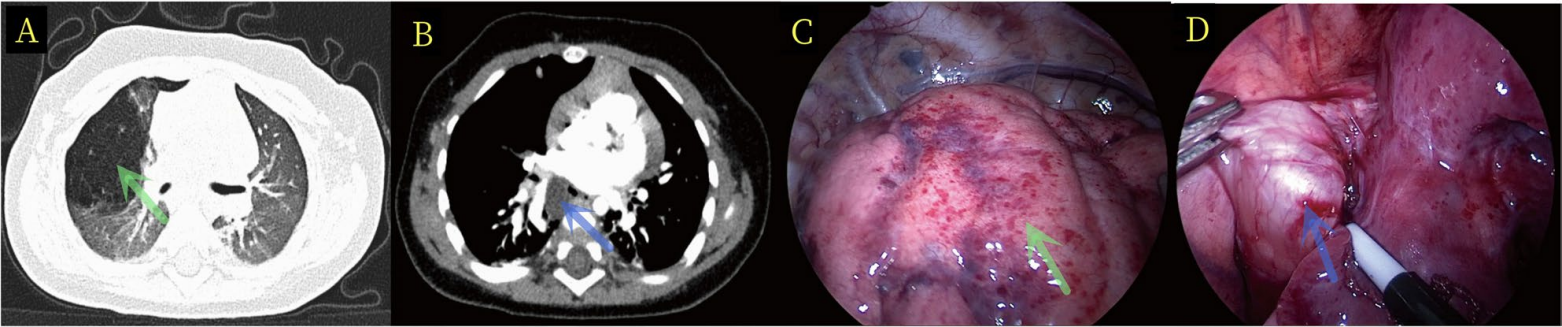
Figure 2 illustrates the preoperative CT scans and intraoperative thoracoscopic observations of Case 6. The CPAM lesions are denoted by green arrows in Fig. 2**A** and 2**C**, whereas the bronchogenic cyst lesions are indicated by blue arrows in Fig. 2**B** and 2**D**

Mediastinal cysts were localized via CT guidance and meticulously dissected along the cyst wall using the electrocautery hook. Cysts with minimal adhesions were enucleated intact. For deeply seated cysts with poor exposure, intentional rupture and aspiration of cystic fluid facilitated complete removal without residual walls. Figure 1 presents the CT examination results and intraoperative thoracoscopy results of Case 1. Fig. 2

## Results

All surgeries were successfully completed thoracoscopically without conversion. Pathological diagnoses included:1.CPAM with bronchogenic cyst (*n* = 7):2.Type 2 CPAM (bronchiolar subtype, *n* = 5)0.3.Type 3 CPAM (bronchiolo-alveolar subtype, *n* = 2).

Operative outcomes: Duration: 90–191 min (median: 110 min).Blood loss: 3–15 mL (median: 10 mL).Chest tube indwelling time: 3–5 days (median: 4 days).During follow-up (median: 29 months; range: 2–59 months), no disease recurrence was observed on chest CT (Table 2 and 3).

**Table 2:** Demonstrates the intraoperative and postoperative manifestations of these seven cases.

Case	Thoracoscopic Procedure	Operative Time (min)	Intraoperative Blood Loss (ml)	Drain Removal Time (days)	Postoperative Pathology	Follow-up Time (months)
1	S6 Segmentectomy	191	10	4	CPAM Type 2	29
2	Anatomical Wedge Resection	101	15	3	CPAM Type 2	54
3	Anatomical Wedge Resection	110	5	4	CPAM Type 2	59
4	Anatomical Wedge Resection	120	10	3	CPAM Type 3	23
5	S3 Segmentectomy	99	5	4	CPAM Type 2	9
6	Anatomical Wedge Resection	180	10	5	CPAM Type 2	2
7	S6 Segmentectomy	90	3	3	CPAM Type 3	32

**Table 3:** Demonstrates the intraoperative and postoperative manifestations of these seven cases.

Case	Thoracoscopic Procedure	Operative Time (min)	Intraoperative Blood Loss (ml)	Drain Removal Time (days)	Postoperative Pathology	CPAM with negative pathological margin	Integrity of bronchogenic cysts	Follow-up Time (months)
1	S6 Segmentectomy	191	10	4	CPAM Type 2	negative	complete	29
2	Anatomical Wedge Resection	101	15	3	CPAM Type 2	negative	complete	54
3	Anatomical Wedge Resection	110	5	4	CPAM Type 2	negative	complete	59
4	Anatomical Wedge Resection	120	10	3	CPAM Type 3	negative	complete	23
5	S3 Segmentectomy	99	5	4	CPAM Type 2	negative	complete	9
6	Anatomical Wedge Resection	180	10	5	CPAM Type 2	negative	complete	2
7	S6 Segmentectomy	90	3	3	CPAM Type 3	negative	complete	32

## Discussion

### Overview of CPAM and bronchogenic cysts

Congenital pulmonary airway malformation (CPAM) is a hamartoma-like cystic lesion caused by abnormal development of lung tissue. CPAM is classified into five subtypes based on gross pathology and histological features: Type 0: Solid appearance, microscopically exhibiting airway wall cartilage, smooth muscle, and glands. Type 1: Cysts measuring 2–10 cm in diameter, lined by pseudostratified ciliated columnar epithelium. Type 2: Cysts 0.5–2 cm in diameter, lined by columnar or cuboidal epithelium. Type 3: Cysts < 0.5 cm in diameter, lined by cuboidal epithelium. Type 4: Cysts > 10 cm in diameter, lined by flattened epithelium [1].Bronchogenic cysts are cystic lesions resulting from abnormal differentiation of the primitive foregut during respiratory tract development [2].With advancements in prenatal diagnostic techniques, the detection rate of CPAM during pregnancy has increased in recent years. In this cohort, six cases were diagnosed prenatally, while one case was identified postnatally via chest CT due to respiratory symptoms (e.g., cough). The current estimated incidence of CPAM is approximately 1.23 per 10,000 live births [5].

The surgical principles of our medical center are as follows: For child patients aged over 3 months and weighing ≥ 4.5 Kg, if the CPAM lesions are detected through imaging CT examination and the parents of the child patients have a strong desire for surgery, surgical treatment is recommended. If conservative treatment is chosen, it is advisable to monitor the respiratory status and the frequency of respiratory diseases, and conduct imaging examinations every 1–2 years to observe the changes in the scope of CPAM lesions. Currently, the parents of all the child patients we have received have opted for surgical treatment. During the follow - up of the child patients after surgery, it has been found that the vital signs of the children are normal and their growth and development are satisfactory.

Among the seven CPAM cases in this group (classified by Stocker’s pathological criteria [1]), five were type 2 and two were type 3.

Thoracoscopic surgery has become the mainstream treatment for CPAM, with surgical approaches evolving from early lobectomy to the current practices of non-anatomical wedge resection or segmentectomy. Given that CPAM is a benign lesion often detected prenatally and associated with favorable prognoses, there is a growing consensus among experts to prioritize lung-preserving techniques (non-anatomical wedge resection or segmentectomy) over lobectomy. This approach retains more functional lung tissue, thereby optimizing long-term pulmonary function [1, 6, 7]. In this series, four patients underwentnon-anatomical wedge resection, and three received segmentectomy.

Bronchogenic cysts, accounting for 5% of primary mediastinal tumors, are cystic masses lined predominantly by respiratory epithelium and arise from abnormal development of the primitive foregut during embryogenesis [2, 8]. While bronchogenic cysts and esophageal duplication cysts share similar anatomical locations, their differentiation relies on histopathology—bronchogenic cysts typically exhibit respiratory epithelial markers (e.g., pseudostratified ciliated columnar epithelium, tracheal cartilage). Surgical excision is the primary treatment for bronchogenic cysts. Complications such as infection or compression of adjacent structures may occur; thus, surgery is recommended when patients exhibit symptoms, parental anxiety exists, or institutional resources permit. The surgical challenge lies in adhesions between the cyst wall and surrounding tissues: severe adhesions may increase operative difficulty and risk of iatrogenic injury [2, 8].

Although numerous hypotheses exist regarding the embryopathological mechanisms of CPAM and bronchogenic cysts, their exact etiology remains unclear. Co-occurrence of these two diseases has been rarely reported. Here, we summarize seven cases of CPAM with concomitant bronchogenic cysts treated via thoracoscopic surgery, aiming to contribute to clinical expertise in this area.

### Imaging examinations

In the imaging diagnosis of congenital pulmonary airway malformation (CPAM) and bronchogenic cysts, chest X-ray and ultrasound serve as essential initial screening tools. Chest CT plays a critical role in the clinical evaluation and management of these two conditions. For CPAM, contrast-enhanced chest CT is the imaging gold standard, enabling precise lesion localization, assessment of lesion extent, cyst size, vascular supply, and mediastinal involvement [1]. In the case of bronchogenic cysts, chest CT is diagnostic in most cases, with CT attenuation values varying depending on cyst content [2, 8].Magnetic resonance imaging (MRI) offers significant advantages in the diagnosis and differential diagnosis of both conditions. However, its application is limited in pediatric patients—particularly young infants—due to high water content interfering with image quality, prolonged scan duration, and high costs [2, 9].In our cohort, all patients underwent preoperative contrast-enhanced chest CT. Notably, small bronchogenic cysts associated with CPAM may be obscured by adjacent pulmonary lesions, potentially leading to preoperative oversight. Fortunately, all seven cases in our study were correctly identified via preoperative CT. When necessary, MRI may be employed for further diagnostic clarification. In cases with high suspicion of concurrent mediastinal cysts, intraoperative exploration should be performed with heightened caution to avoid missing lesions.

### Experience with one-stage surgery for ipsilateral intrathoracic combined lesions

Advantages of one-stage surgery for ipsilateral intrathoracic combined lesions: The number of incisions is reduced; secondary anesthesia is avoided; the trauma and psychological distress associated with a second surgery are eliminated; medical resources and costs are saved. Disadvantages: Incision selection can be challenging; the duration of anesthesia and surgery is longer compared to a single procedure; surgical risks are increased. For one-stage surgery, it is essential to maintain stable respiratory and circulatory functions and minimize the total duration of anesthesia and surgery. During the operation, gentle manipulation should be practiced to reduce repeated excessive clamping of lung tissue and other organs, thereby preventing pulmonary edema, hemorrhage, and contusion [10–15].Our treatment principle prioritized pulmonary lesions, addressing them first before managing bronchogenic cysts. In all seven cases included in this study, the bronchogenic cysts were located near the CPAM lesions, with the two sites in close proximity, making incision selection relatively straightforward. However, if the lesions are far apart in clinical practice, the surgical approach may be adjusted by adding appropriately placed trocars to facilitate the operation. For pulmonary surgical wounds, we used 4 − 0 PDS sutures to minimize air leakage. All seven cases underwent one-stage surgery with standard operative techniques, and no intraoperative complications occurred. Therefore, one-stage surgery is safe and feasible, though it carries certain risks. In the seven reported cases, the bronchogenic cysts were easily identifiable during surgery, with minimal adhesions to surrounding tissues. The dissection was performed along the cyst wall. If the cyst was deeply located and its margins were poorly exposed, the cyst was intentionally ruptured, and the fluid was aspirated before further manipulation. All seven pediatric cases had smooth bronchogenic cyst resections without residual cyst walls. If a bronchogenic cyst shares a large common wall with the trachea, making dissection difficult, piecemeal resection may be performed. In cases where complete cyst removal is challenging, subtotal resection can be carried out, with the remaining cyst wall mucosa ablated using electrocautery or iodine to prevent recurrence [2, 8].

### Postoperative observation and follow-up

For patients undergoing one-stage surgery for two ipsilateral intrathoracic lesions, close monitoring of vital signs is essential postoperatively. In this cohort, all 7 cases were transferred to the general ward after surgery, where enhanced analgesia, nebulization, positional changes with back percussion for sputum expulsion, and close observation of drainage tubes (for bleeding or air leakage) were implemented. Pulmonary status was assessed via auscultation of breath sounds or chest radiography.

All 7 cases underwent routine non-contrast chest CT scans at 3 months postoperatively. If no abnormalities were detected, follow-up scans were recommended every 36 months. None of the cases developed postoperative pneumothorax or recurrence, with all achieving favorable recovery. Abnormal findings would necessitate shortened follow-up intervals and targeted interventions based on clinical evaluation.

### Study limitations and future perspectives

Sample size limitation: This study reported only 7 pediatric cases of congenital pulmonary airway malformation (CPAM) coexisting with bronchogenic cysts, representing a significant limitation compared to large-scale studies.

Surgical approach limitation: In our cohort, the CPAM and bronchogenic cysts were anatomically proximate, allowing unified trocar placement during thoracoscopic surgery centered on the CPAM lesion. However, optimal trocar positioning for widely separated lesions remains a technical challenge, highlighting a limitation of our approach.

Intraoperative feasibility limitation: The bronchogenic cysts in these 7 cases were easily localized intraoperatively with minimal adhesions, facilitating resection. Management strategies for cysts with severe perilesional adhesions remain unaddressed, underscoring another constraint of this study. Future multicenter collaborative studies may help overcome these limitations.

## Conclusion

CPAM coexisting with bronchogenic cysts is rare in children. Meticulous preoperative imaging and intraoperative exploration are critical to avoid missed diagnoses. Thoracoscopic surgery is a method that can be attempted for treating CPAM in children with ipsilateral mediastinal bronchogenic cysts. 

## Supplementary Information

Supplementary Material 1.

Supplementary Material 2.

## Data Availability

Data is provided within the manuscript.

## Abbreviations

CPAM: Congenital Pulmonary Airway Malformation
CT: Computed Tomography
MRI: Magnetic Resonance Imaging
