# Supplementary material for: Unicentric report on thoracoscopic surgery 7 cases for congenital pulmonary airway malformation combined with ipsilateral mediastinal bronchogenic cyst in children
Source: BMC Surg. 2026 Mar 5;26:241. doi: 10.1186/s12893-026-03617-5 (PMC13045126; doi:10.1186/s12893-026-03617-5)
Supplement: Supplementary file 2 — Supplementary Material 2. [file 12893_2026_3617_MOESM2_ESM.docx]

| **Case** | **Gender** | **Age (Months)** | **Lesion Location** | **Clinical Presentation** | **CPAM position** | **Location of mediastinal cyst** | **Size of Mediastinal Cyst (cm)** | **Thoracoscopic Procedure** | **Operative Time (min)** | **Intraoperative Blood Loss (ml)** | **Drain Removal Time (days)** | **Postoperative Pathology** | **CPAM with negative pathological margin** | **Integrity of bronchogenic cysts** | **Follow-up Time (months)** |
| --- | --- | --- | --- | --- | --- | --- | --- | --- | --- | --- | --- | --- | --- | --- | --- |
| 1 | Male | 4 | Right | Detected during prenatal ultrasound at 23 weeks of gestation | S6 | The lower pulmonary ligament and beside the esophagus | 1.2 × 1.6 × 1.3 | S6 Segmentectomy | 191 | 10 | 4 | CPAM Type 2 | negative | complete | 29 |
| 2 | Male | 6 | Right | Detected during prenatal ultrasound at 28 weeks of gestation | Localized lower lobe of the lung | Between the inferior pulmonary vein and the lower lobe bronchi | 1.8 × 1.3 × 1.0 | Non-Anatomical Wedge Resection | 101 | 15 | 3 | CPAM Type 2 | negative | complete | 54 |
| 3 | Male | 7 | Right | Detected during prenatal ultrasound at 23 weeks of gestation | Localized lower lobe of the lung | The pleura on the lateral side of the lower pulmonary ligament | 1.8 × 1.5 × 1.0 | Non-Anatomical Wedge Resection | 110 | 5 | 4 | CPAM Type 2 | negative | complete | 59 |
| 4 | Female | 67 | Right | Detected on chest CT due to cough lasting 20 days | Localized lower lobe of the lung | The origin of the right pulmonary oblique fissure | 4.1 × 2.1 × 2.6 | Non-Anatomical Wedge Resection | 120 | 10 | 3 | CPAM Type 3 | negative | complete | 23 |
| 5 | Female | 7 | Left | Detected during prenatal ultrasound at 22 weeks of gestation | S3 | Between the left main bronchus and the thoracic aorta | 1.1 × 1.1 × 0.6 | S3 Segmentectomy | 99 | 5 | 4 | CPAM Type 2 | negative | complete | 9 |
| 6 | Male | 5 | Right | Detected during prenatal ultrasound at 24 weeks of gestation | Localized upper lobe of the lung | Below the strange vein, between the esophagus and the hilum of the lung | 1.2 × 1.2 × 1.4 | Non-Anatomical Wedge Resection | 180 | 10 | 5 | CPAM Type 2 | negative | complete | 2 |
| 7 | Male | 20 | Right | Detected during prenatal ultrasound at 28 weeks of gestation | S6 | Between the inferior pulmonary vein and the lower lobe bronchi | 1.1 × 1.1 × 0.6 | S6 Segmentectomy | 90 | 3 | 3 | CPAM Type 3 | negative | complete | 32 |
