# Supplementary material for: Unicentric report on thoracoscopic surgery 7 cases for congenital pulmonary airway malformation combined with ipsilateral mediastinal bronchogenic cyst in children
Source: BMC Surg. 2026 Mar 5;26:241. doi: 10.1186/s12893-026-03617-5 (PMC13045126; doi:10.1186/s12893-026-03617-5)
Supplement: Supplementary file 1 — Supplementary Material 1. [file 12893_2026_3617_MOESM1_ESM.doc]

[Insert the date of submission]

June 6, 2025

Jieying Zhou, PhD

Associate Editor

*BMC Pediatrics*

Dear Editor:

We/I wish to submit a treatise for publication in

*BMC Pediatrics*, titled “Unicentric Report on Thoracoscopic Surgery for Congenital Pulmonary Airway Malformation Combined with Ipsilateral Mediastinal Bronchogenic Cyst in Children

**.” The paper was co-authored by Huashan Zhao,Shumin Zhao,Yungpeng Zhai,Rui Guo,Gang Shen,Hongxiu Xu,Sai Huang，Shisong Zhang.**

From January 2019 to January 2025, 7 cases of congenital pulmonary airway malformation (CPAM) combined with bronchogenic cyst were treated with thoracoscopic surgery. A three-port approach was adopted via the lateral chest. The patients were placed in a lateral decubitus position with the healthy side down. The observation port was established at the intersection of the scapular line and the 5th intercostal space, while the operating ports were created based on surgical needs using the diamond-shaped method, with all ports utilizing 5 mm trocars. CO2 pneumothorax was maintained at a pressure of 4 mmHg and a flow rate of 2 L/min, with adjustments made according to intraoperative conditions. The surgical incision was primarily designed for pulmonary surgery, with lung surgery performed first, followed by resection of the bronchogenic cyst. Piecemeal resection was employed when necessary to avoid severe complications related to the trachea.CPAM or pulmonary sequestration in children may coexist with bronchogenic cysts. Preoperative imaging should be carefully correlated with intraoperative findings to avoid missed diagnoses. Thoracoscopic surgery is a safe and feasible approach for the treatment of CPAM or pulmonary sequestration combined with ipsilateral mediastinal bronchogenic cyst in children.

Further, we believe that this paper will be of interest to the readership of your journal, as it presents and addresses challenges in pediatric patient care and child health, particularly in the diagnosis and management of rare congenital pulmonary anomalies.

This manuscript has not been published or presented elsewhere in part or entirety and is not under consideration by another journal. All study participants provided informed consent, and the study design was approved by the appropriate ethics review board.We have read and understood your journal’s policies, and we believe that neither the manuscript nor the study violates any of these. There are no conflicts of interest to declare.

Thank you for your consideration. I look forward to hearing from you.

Sincerely,

[Author's name] Huashan Zhao

[Affiliation] Department of Thoracic and Oncological Surgery, Children’s Hospital Affiliated to Shandong University(Jinan Children’s Hospital), 23976 Jingshi Road, Huaiyin District, Jinan 250022, China.

[Postal address] Department of Thoracic and Oncological Surgery, Children’s Hospital Affiliated to Shandong University(Jinan Children’s Hospital), 23976 Jingshi Road, Huaiyin District, Jinan 250022, China.

[Phone number]13869103697

[Fax number]13869103697

[Email address] zhsly1987@126.com
